# Supplementary material for: Mutant p53 Mediates Sensitivity to Cancer Treatment Agents in Oesophageal Adenocarcinoma Associated with MicroRNA and SLC7A11 Expression
Source: Int J Mol Sci. 2021 May 24;22(11):5547. doi: 10.3390/ijms22115547 (PMC8197322; doi:10.3390/ijms22115547)
Supplement: Supplementary file 1 [file ijms-22-05547-s001.zip › ijms-1186325-supplementary/ijms-1186325 supplementary.v4/Supplementary Data revised/S1 results revised.pdf]

Supplementary Data 1:

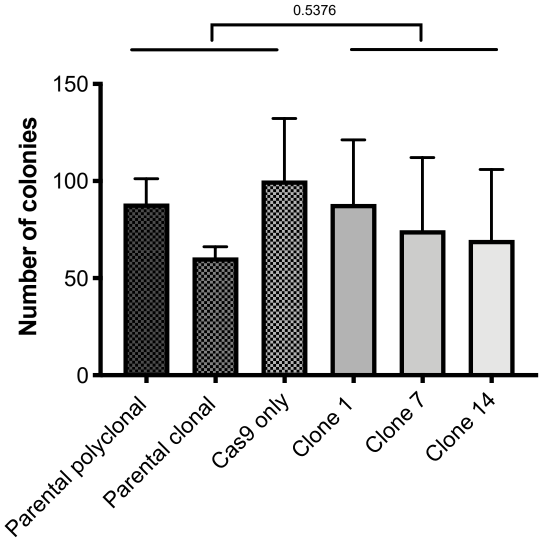

Supplementary Figure 1. Colony formation of mock-irradiated Parental (Parental polyclonal, Parental clonal, Cas9 only) and p53-KO (Clone 1, Clone 7 and Clone 14) cells. There was no difference in the ability of mock-irradiated Parentals and p53-KO cells to form colonies (p = 0.538

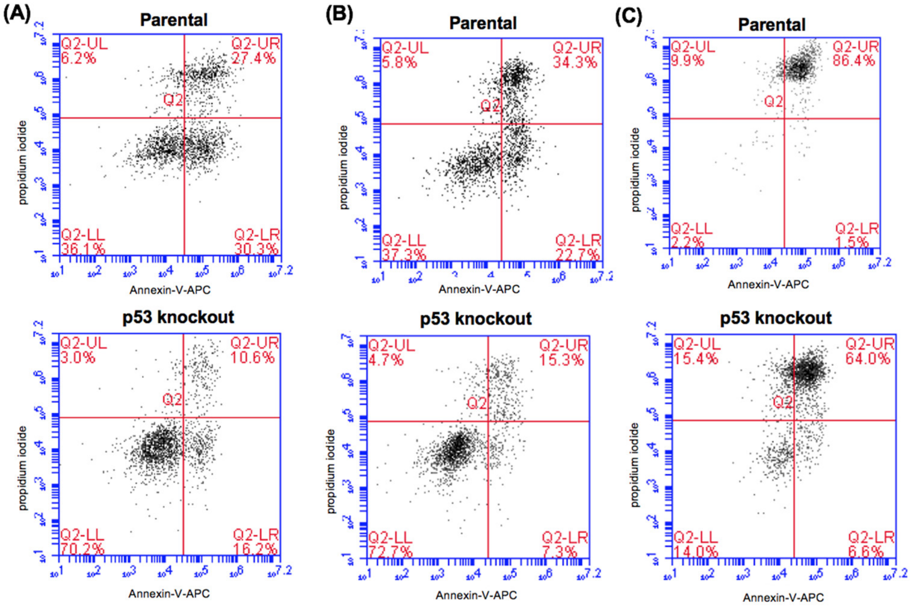

Supplementary Figure 2. Examples of the Annexin-V-APC apoptosis assays. Examples of the Annexin-V-APC apoptosis assay following (A) 20 µM 5-FU treatment, (B) 10 µM 4-hydroxytamoxifen treatment, and (C) 10 µM endoxifen treatment. The population of p53-KO viable cells (lower left quadrant) following all treatments was higher than the Parental population while the (early and late) apoptotic population was higher for the Parental cells.

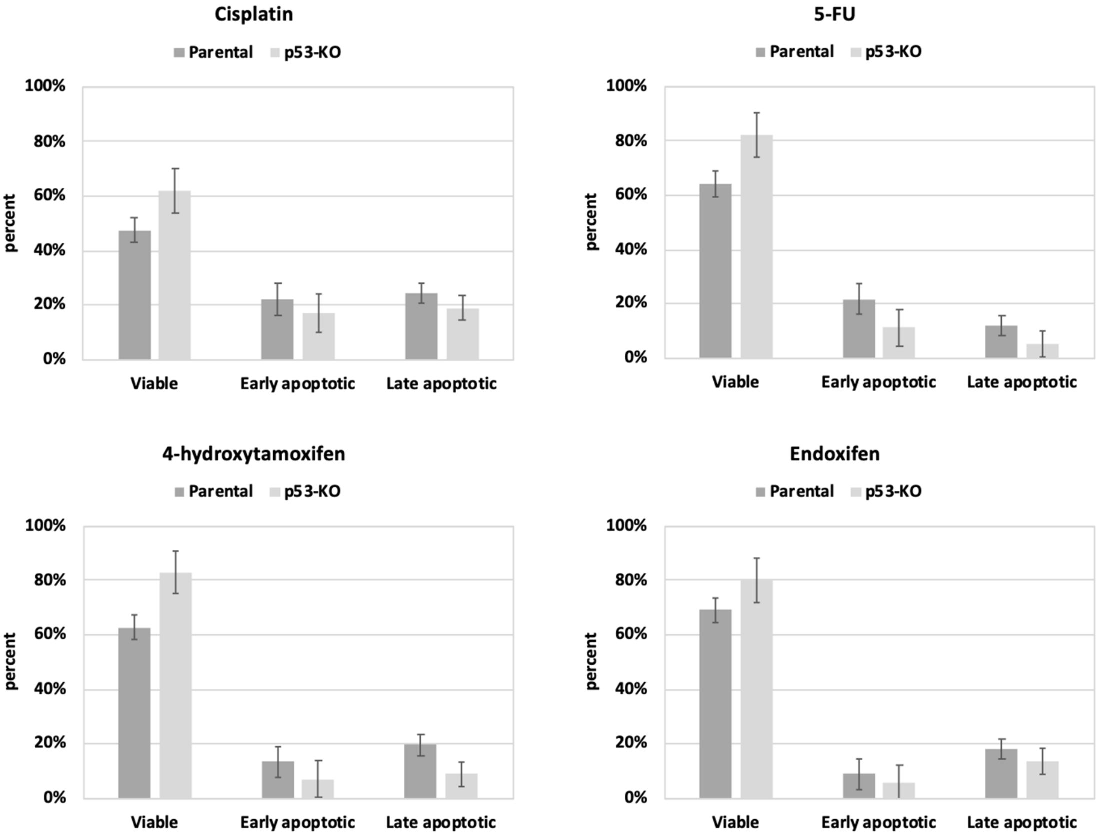

Supplementary Figure 3. Responses of Parental and p53-KO cells to drug treatments. Cells were assessed using the Annexin-V-APC apoptosis assay following 72 h-treatment with either 8 μM cisplatin, 19 μM 5-FU, 7.8 μM 4-hydroxytamoxifen, or 6.7 μM endoxifen.

Supplementary Table 1a. Gene Ontology Selection of terms in which the validated targets of miR-27a-3p were significantly enriched

Gene Ontology Term	Pathway uploaded gene count	Genes in InnateDB for this entity	Pathway p-value (corrected)
Regulation of transcription, DNA-templated	89	1898	8.36×10^{-10}
Apoptotic process	33	639	5.24×10^{-4}
Cell cycle arrest	13	128	5.77×10^{-4}
Gene expression	33	680	0.001
Cellular response to DNA damage stimulus	15	192	0.002
Negative regulation of cell proliferation	22	406	0.005
Positive regulation of autophagy	5	22	0.006
Negative regulation of apoptotic process	25	501	0.006
Positive regulation of apoptotic process	18	307	0.007

Regulation of cell proliferation	12	156	0.008
Wnt signalling pathway	11	170	0.034
Signal transduction in response to DNA damage	3	11	0.035
Intrinsic apoptotic signalling pathway by p53 class mediator	4	24	0.038
Cellular response to ionizing radiation	4	26	0.045

Supplementary Table 1b. Biological Pathway Selection of pathways in which the validated targets of miR-27a-3p were significantly enriched

Pathway Name	Pathway uploaded gene count	Genes in InnateDB for this entity	Pathway p-value (corrected)
Fatty acid, triacylglycerol and ketone body metabolism	16	176	0.002
EGFR1	28	472	0.002
Direct p53 effectors	12	129	0.004
Wnt signalling pathway	10	91	0.004
Regulation of lipid metabolism by Peroxisome proliferator-activated receptor alpha (PPARalpha)	11	109	0.004
Nuclear Receptor transcription pathway	6	39	0.01
Metabolism of lipids and lipoproteins	28	554	0.010
p53 pathway	6	47	0.017
Cell cycle	10	126	0.018
Pyruvate metabolism	4	24	0.029
Rb tumour suppressor/checkpoint signalling in response to DNA damage	3	13	0.033
Gene Expression	43	1118	0.034

Supplementary Table 2a. Gene Ontology Selection of terms in which the validated targets of all increased miRNAs were significantly enriched

Gene Ontology Term	Pathway uploaded gene count	Genes in InnateDB for this entity	Pathway p-value (corrected)
Cellular response to hypoxia	42	97	8.39×10^{-12}
Cell cycle arrest	46	128	1.43×10^{-9}
Gene expression	145	680	2.19×10^{-9}
Regulation of cell cycle	45	137	4.87×10^{-8}
Apoptotic process	129	639	6.89×10^{-7}
Cellular response to DNA damage stimulus	53	192	1.13×10^{-6}
Positive regulation of cell proliferation	96	462	1.35×10^{-5}
Intrinsic apoptotic signalling pathway	23	58	1.64×10^{-5}
Cell proliferation	79	370	4.78×10^{-5}
Stress-activated MAPK cascade	21	54	6.28×10^{-5}
Negative regulation of apoptotic process	98	501	1.27×10^{-4}
Regulation of cell proliferation	40	156	2.93×10^{-4}
Response to drug	64	313	0.001
Cell growth	18	52	0.002
Cell activation	7	10	0.002

Regulation of apoptotic process	43	198	0.006
Response to gamma radiation	12	30	0.006
Cell cycle	46	217	0.006
Positive regulation of autophagy	10	22	0.006
Negative regulation of translation involved in gene silencing by miRNA	6	9	0.009
p53 binding	17	56	0.010
Regulation of autophagy	9	20	0.012
Signal transduction	208	1368	0.013
Epithelial to mesenchymal transition	12	34	0.016
Negative regulation of glycolytic process	5	7	0.016
Intrinsic apoptotic signalling pathway in response to DNA damage by p53 class mediator	11	30	0.018
Cellular response to ionizing radiation	10	26	0.020
DNA damage checkpoint	11	31	0.023
Cellular lipid metabolic process	33	154	0.025
Response to toxic substance	20	78	0.025
Cellular response to drug	15	52	0.028
Intrinsic apoptotic signalling pathway in response to DNA damage	16	58	0.031
Glucose metabolic process	27	121	0.032
Intrinsic apoptotic signalling pathway by p53 class mediator	9	24	0.035
Negative regulation of oxidative stress-induced intrinsic apoptotic signalling pathway	6	12	0.038
Positive regulation of DNA damage response. signal transduction by p53 class mediator	6	12	0.038
Regulation of miRNA metabolic process	3	3	0.041
DNA replication	32	155	0.041
Positive regulation of fatty acid beta-oxidation	5	9	0.047
DNA damage response. signal transduction by p53 class mediator resulting in cell cycle arrest	17	67	0.048

Supplementary Table 2b. Biological Pathway Selection of pathways in which the validated targets of all increased miRNAs were significantly enriched

Pathway Name	Pathway uploaded gene count	Genes in InnateDB for this entity	Pathway p-value (corrected)
Cellular responses to stress	81	240	1.93 x 10^{-12}
Pathways in cancer	94	329	8.30 x 10^{-10}
p53 pathway	25	47	4.20 x 10^{-8}
p53 signalling pathway	31	68	4.45 x 10^{-8}
Gene Expression	223	1118	4.13 x 10^{-7}
Cell cycle	42	126	1.30 x 10^{-6}
Cyclins and cell cycle regulation	14	23	8.72 x 10^{-6}
Direct p53 effectors	40	129	1.61 x 10^{-5}

Rb tumour suppressor/checkpoint signalling in response to DNA damage	10	13	1.75×10^{-5}
Wnt signalling pathway	42	140	2.08×10^{-5}
Cellular response to hypoxia	13	25	1.72×10^{-4}
Fatty acid, triacylglycerol and ketone body metabolism	46	176	2.61×10^{-4}
mTOR signalling pathway	22	62	3.03×10^{-4}
Regulation of lipid metabolism by Peroxisome proliferator-activated receptor alpha (PPARalpha)	32	109	3.92×10^{-4}
VEGF signalling pathway	46	183	6.06×10^{-4}
TGF-beta signalling pathway	25	80	8.39×10^{-4}
Signal Transduction	4	4	0.004
Intrinsic Pathway for Apoptosis	13	38	0.008
Pten dependent cell cycle arrest and apoptosis	7	15	0.013
Apoptosis	35	155	0.013
Plasma membrane oestrogen receptor signalling	9	24	0.019
Signalling to RAS	10	31	0.032
Apoptotic signalling in response to DNA damage	6	14	0.034
Metabolism of lipids and lipoproteins	97	554	0.040
p53-Dependent G1 DNA Damage Response	15	57	0.040
p53-Dependent G1/S DNA damage checkpoint	15	57	0.040

Supplementary Table 3a. Gene Ontology Selection of Terms in which the validated targets of miR-324-3p were significantly enriched

Gene Ontology Term	Pathway uploaded gene count	Genes in InnateDB for this entity	Pathway p-value (corrected)
Poly(A) RNA binding	63	1104	5.94×10^{-14}
Protein binding	235	9632	4.92×10^{-12}
Cytosol	86	2642	1.65×10^{-6}
Membrane	96	3199	7.07×10^{-6}
Nucleus	145	5730	1.89×10^{-5}
Ribonucleoprotein complex	14	133	3.11×10^{-5}
Cellular protein metabolic process	28	538	5.65×10^{-5}
Translation	19	274	6.51×10^{-5}
Gene expression	32	680	6.64×10^{-5}
Cytoplasm	122	4897	4.08×10^{-4}
Translational termination	10	92	5.77×10^{-4}
SRP-dependent cotranslational protein targeting to membrane	11	114	5.87×10^{-4}
RNA binding	25	546	0.001

Supplementary Table 3b. Biological Pathway Selection of pathways in which the validated targets of miR-324-3p were significantly enriched

Pathway Name	Pathway uploaded gene count	Genes in InnateDB for this entity	Pathway p-value (corrected)
Translation	15	145	1.61×10^{-4}

Peptide chain elongation	12	84	2.01×10^{-4}
GTP hydrolysis and joining of the 60S ribosomal subunit	11	105	8.71×10^{-4}
Metabolism of proteins	29	678	0.014
Ribosome	10	137	0.029

Supplementary Table 4. Gene Ontology Selection of terms in which the validated targets of all decreased miRNAs were significantly enriched

Pathway Name	Pathway uploaded gene count	Genes in InnateDB for this entity	Pathway p-value (corrected)
Protein binding	2052	9632	1.44×10^{-43}
Cytosol	717	2642	2.99×10^{-38}
Nucleus	1323	5730	3.57×10^{-37}
Poly(A) RNA binding	360	1104	1.43×10^{-33}
Cytoplasm	1146	4897	5.28×10^{-33}
Nucleoplasm	352	1132	2.89×10^{-28}
Gene expression	241	680	1.26×10^{-27}
Membrane	743	3199	1.35×10^{-17}
Mitotic cell cycle	141	416	1.17×10^{-13}
Translation	102	274	1.94×10^{-12}
Cellular protein metabolic process	164	538	1.95×10^{-11}
RNA metabolic process	93	254	7.46×10^{-11}
mRNA metabolic process	86	228	8.48×10^{-11}
Regulation of apoptotic process	69	198	5.89×10^{-7}
DNA repair	96	311	8.42×10^{-7}
Protein folding	63	178	1.30×10^{-6}
Regulation of cell cycle	51	137	4.87×10^{-6}
Regulation of cell proliferation	54	156	2.64×10^{-5}
Apoptotic process	162	639	2.85×10^{-5}
DNA replication	53	155	4.89×10^{-5}
Cell proliferation	102	370	8.53×10^{-5}
DNA damage response, signal transduction by p53 class mediator resulting in cell cycle arrest	29	67	9.02×10^{-5}
Cellular response to DNA damage stimulus	60	192	2.20×10^{-4}
Ribosome	56	178	3.42×10^{-4}
Cellular metabolic process	50	153	3.44×10^{-4}
Response to drug	86	313	5.39×10^{-4}
Positive regulation of epithelial to mesenchymal transition	13	26	0.008
Small ribosomal subunit	13	26	0.008
Regulation of Ras protein signal transduction	6	7	0.009
Intrinsic apoptotic signalling pathway	22	58	0.009
Cell cycle	59	217	0.011
Membrane organization	44	152	0.014
Wnt signalling pathway	48	170	0.015
Protein phosphorylation	143	630	0.016
Cell division	29	90	0.020
Negative regulation of extrinsic apoptotic signalling pathway	16	39	0.020
Positive regulation of DNA replication	16	39	0.020

Response to radiation	14	32	0.020
Alpha-linolenic acid metabolic process	7	11	0.029
Intrinsic apoptotic signalling pathway in response to DNA damage by p53 class mediator	13	30	0.029
Glucose metabolic process	35	121	0.037
Negative regulation of DNA damage response, signal transduction by p53 class mediator	7	12	0.048
