# Supplementary material for: Mutant p53 Mediates Sensitivity to Cancer Treatment Agents in Oesophageal Adenocarcinoma Associated with MicroRNA and SLC7A11 Expression
Source: Int J Mol Sci. 2021 May 24;22(11):5547. doi: 10.3390/ijms22115547 (PMC8197322; doi:10.3390/ijms22115547)

Supplementary Data 3:

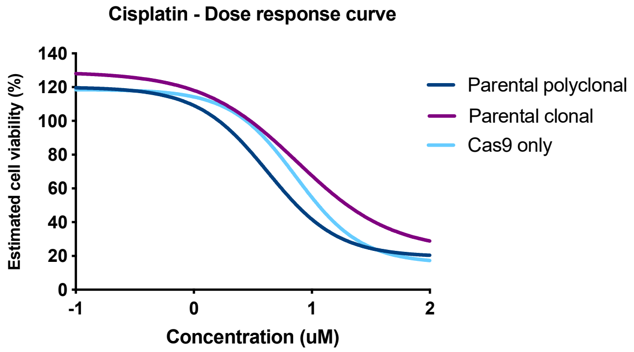

Supplementary Figure 1. Cisplatin dose response The Dose response curves were generated by MTS assay. The individual Parental cell lines (Parental polyclonal, Parental clonal and Cas9 only) demonstrated similar MTS dose response curves.

Supplementary Table 1. Details of House Keeping Gene miRNAs selected from the p53-KO and Parental JHEso-Ad1 cell lines.

OpenArray ID	miRBase v22 sequence	miRBase v22 ID	miRBase Accession
000407_hsa-miR-26b	UUCAAGUAAUUCAGGAUAGGU	hsa-miR-26b-5p	MIMAT0000083
000405_hsa-miR-26a	UUCAAGUAAUCCAGGAUAGGCU	hsa-miR-26a-5p	MIMAT0000082
002282_hsa-let-7g	UGAGGUAGUAGUUUGUACAGUU	hsa-let-7g-5p	MIMAT0000414
000442_hsa-miR-106b	UAAAGUGCUGACAGUGCAGAU	hsa-miR-106b-5p	MIMAT0000680
001515_hsa-miR-660	UACCCAUUGCAUAUCGGAGUUG	hsa-miR-660-5p	MIMAT0003338
000563_hsa-miR-374	UUAUAAUACAACCUGAUAAGUG	hsa-miR-374a-5p	MIMAT0000727
002422_hsa-miR-18a	UAAGGUGCAUCUAGUGCAGAUAG	hsa-miR-18a-5p	MIMAT0000072
000545_hsa-miR-331	GCCCCUGGGCCUAUCCUAGAA	hsa-miR-331-3p	MIMAT0000760
001518_hsa-miR-532	CAUGCCUUGAGUGUAGGACCGU	hsa-miR-532-5p	MIMAT0002888
000470_hsa-miR-148a	UCAGUGCACUACAGAACUUUGU	hsa-miR-148a-3p	MIMAT0000243
000408_hsa-miR-27a	UUCACAGUGGCUAAGUUCCGC	hsa-miR-27a-3p	MIMAT0000084
002258_hsa-miR-340	UUAUAAAGCAAUGAGACUGAUU	hsa-miR-340-5p	MIMAT0004692
002271_hsa-miR-185	UGGAGAGAAAGGCAGUUCCUGA	hsa-miR-185-5p	MIMAT0000455
000493_hsa-miR-194	UGUAACAGCAACUCCAUGUGGA	hsa-miR-194-5p	MIMAT0000460
000420_hsa-miR-30d	UGUAAACAUCCCCGACUGGAAG	hsa-miR-30d-5p	MIMAT0000245
001090_mmu-miR-93	CAAAGUGCUGUUCGUGCAGGUAG	mmu-miR-93-5p	MIMAT0000540
002367_hsa-miR-193b	AACUGGCCCUCAAAGUCCCGCU	hsa-miR-193b-3p	MIMAT0002819
000494_hsa-miR-195	UAGCAGCACAGAAAUAUUGGC	hsa-miR-195-5p	MIMAT0000461
000507_hsa-miR-203	GUGAAAUGUUUAGGACCACUAG	hsa-miR-203a-3p	MIMAT0000264
000417_hsa-miR-30a-5p	UGUAAACAUCCUCGACUGGAAG	hsa-miR-30a-5p	MIMAT0000087
000411_hsa-miR-28	AAGGAGCUCACAGUCUAUUGAG	hsa-miR-28-5p	MIMAT0000085
002324_hsa-miR-744	UGCGGGGCUAGGGCUAACAGCA	hsa-miR-744-5p	MIMAT0004945
002276_hsa-miR-222	AGCUACAUCUGGCUACUGGGU	hsa-miR-222-3p	MIMAT0000279
002883_hsa-miR-1274A	GUCCCUGUUCAGGCGCCA	hsa-miR-1274A	MI0006410
002349_hsa-miR-574-3p	CACGCUCAUGCACACACCCACA	hsa-miR-574-3p	MIMAT0003239
002187_hsa-miR-942	UCUUCUCUGUUUUGGCCAUGUG	hsa-miR-942-5p	MIMAT0004985
000539_hsa-miR-324-5p	CGCAUCCCCUAGGGCAUUGGUG	hsa-miR-324-5p	MIMAT0000761
002446_hsa-miR-28-3p	CACUAGAUUGUGAGCUCCUGGA	hsa-miR-28-3p	MIMAT0004502

000403_hsa-miR-25	CAUUGCACUUGUCUCGGUCUGA	hsa-miR-25-3p	MIMAT0000081
000397_hsa-miR-21	UAGCUUAUCAGACUGAUGUUGA	hsa-miR-21-5p	MIMAT0000076
000518_hsa-miR-215	AUGACCUAUGAAUUGACAGAC	hsa-miR-215-5p	MIMAT0000272
000475_hsa-miR-152	UCAGUGCAUGACAGAACUUGG	hsa-miR-152-3p	MIMAT0000438
001986_hsa-miR-766	ACUCCAGCCCCACAGCCUCAGC	hsa-miR-766-3p	MIMAT0003888
001097_hsa-miR-146b	UGAGAACUGAAUUCCAUAGGCUG	hsa-miR-146b-5p	MIMAT0002809
000456_hsa-miR-130b	CAGUGCAAUGAUGAAAGGGCAU	hsa-miR-130b-3p	MIMAT0000691
000546_hsa-miR-335	UCAAGAGCAAUAACGAAAAAUGU	hsa-miR-335-5p	MIMAT0000765
002277_hsa-miR-320	AAAAGCUGGGUUGAGAGGGCGA	hsa-miR-320a-3p	MIMAT0000510
002884_hsa-miR-1274B	UCCCUGUUCGGGCGCCA	hsa-miR-1274B	MI0006427
002365_hsa-miR-494	UGAAACAUACACGGGAAACCUC	hsa-miR-494-3p	MIMAT0002816
002289_hsa-miR-139-5p	UCUACAGUGCACGUGUCUCCAGU	hsa-miR-139-5p	MIMAT0000250
002355_hsa-miR-532-3p	CCUCCCACACCCAAGGCUUGCA	hsa-miR-532-3p	MIMAT0004780
000564_hsa-miR-375	UUUGUUCGUUCGGCUCGCGUGA	hsa-miR-375-3p	MIMAT0000728
000387_hsa-miR-10a	UACCCUGUAGAUCCGAAUUUGUG	hsa-miR-10a-5p	MIMAT0000253
000524_hsa-miR-221	AGCUACAUUGUCUGCUGGGUUUC	hsa-miR-221-3p	MIMAT0000278
001020_hsa-miR-365	UAAUGCCCCUAAAAAUCCUUAU	hsa-miR-365a-3p	MIMAT0000710
002340_hsa-miR-423-5p	UGAGGGGCAGAGAGCGAGACUUU	hsa-miR-423-5p	MIMAT0004748
000449_hsa-miR-125b	UCCCUGAGACCCUAACUUGUGA	hsa-miR-125b-5p	MIMAT0000423
002838_hsa-miR-1291	UGGCCCUGACUGAAGACCAGCAGU	hsa-miR-1291	MIMAT0005881

Supplementary Table 2. Details of miRNAs that were differentially expressed after p53-KO

Open Array identifier	Open Array assay number	Open Array sequence	miRBase v22 sequence	miRBase v22 Identifier	miRBase Accession number
002161_hsa-miR-324-3p	2161	ACUGCCCCAGGUGCUGCUGG	CCCACUGCCCCAGGUGCUGCUGG	hsa-miR-324-3p	MIMAT0000762
000408_hsa-miR-27a	408	UUCACAGUGGCUAAGUUCCGC	UUCACAGUGGCUAAGUUCCGC	hsa-miR-27a-3p	MIMAT0000084
000402_hsa-miR-24	402	UGGCUCAGUUCAGCAGGAACAG	UGGCUCAGUUCAGCAGGAACAG	hsa-miR-24-3p	MIMAT0000080
002186_hsa-miR-345	2186	GCUGACUCCUAGUCCAGGGCUC	GCUGACUCCUAGUCCAGGGCUC	hsa-miR-345-5p	MIMAT0000772
000543_hsa-miR-328	543	CUGGCCCUCUCUGCCCUUCCGU	CUGGCCCUCUCUGCCCUUCCGU	hsa-miR-328-3p	MIMAT0000752
002271_hsa-miR-185	2271	UGGAGAGAAAGGCAGUUCCUGA	UGGAGAGAAAGGCAGUUCCUGA	hsa-miR-185-5p	MIMAT0000455
001097_hsa-miR-146b	1097	UGAGAACUGAAUUCCAUAGGCU	UGAGAACUGAAUUCCAUAGGCUG	hsa-miR-146b-5p	MIMAT0002809
000512_hsa-miR-210	512	CUGUGCGUGUGACAGCGGCUGA	CUGUGCGUGUGACAGCGGCUGA	hsa-miR-210-3p	MIMAT0000267
000456_hsa-miR-130b	456	CAGUGCAAUGAUGAAAGGGCAU	CAGUGCAAUGAUGAAAGGGCAU	hsa-miR-130b-3p	MIMAT0000691
002234_hsa-miR-140-3p	2234	UACCACAGGGUAGAACCACGG	UACCACAGGGUAGAACCACGG	hsa-miR-140-3p	MIMAT0004597
000405_hsa-miR-26a	405	UUCAAGUAAUCCAGGAUAGGCU	UUCAAGUAAUCCAGGAUAGGCU	hsa-miR-26a-5p	MIMAT0000082
000539_hsa-miR-324-5p	539	CGCAUCCCCUAGGGCAUUGGUGU	CGCAUCCCCUAGGGCAUUGGUG	hsa-miR-324-5p	MIMAT0000761
000480_hsa-miR-181a	480	AACAUUCAACGCUGUCGGUGAGU	AACAUUCAACGCUGUCGGUGAGU	hsa-miR-181a-5p	MIMAT0000256
000407_hsa-miR-26b	407	UUCAAGUAAUUCAGGAUAGGU	UUCAAGUAAUUCAGGAUAGGU	hsa-miR-26b-5p	MIMAT0000083
002277_hsa-miR-320	2277	AAAAGCUGGGUUGAGAGGGCGA	AAAAGCUGGGUUGAGAGGGCGA	hsa-miR-320a-3p	MIMAT0000510

Supplementary Table 3. Details of House Keeping Gene miRNAs selected from the SLC7A11 knockdown experiments.

OpenArray ID	miRBase v22 sequence	miRBase v22 ID	miRBase Accession
000405_hsa-miR-26a	UUCAAGUAAUCCAGGAUAGGCU	hsa-miR-26a-5p	MIMAT0000082
000407_hsa-miR-26b	UUCAAGUAAUUCAGGAUAGGU	hsa-miR-26b-5p	MIMAT0000083
002422_hsa-miR-18a	UAAGGUGCAUCUAGUGCAGAUAG	hsa-miR-18a-5p	MIMAT0000072
002282_hsa-let-7g	UGAGGUAGUAGUUUGUACAGUU	hsa-let-7g-5p	MIMAT0000414
001518_hsa-miR-532	CAUGCCUUGAGUGUAGGACCGU	hsa-miR-532-5p	MIMAT0002888
000411_hsa-miR-28	AAGGAGCUCACAGUCUAUUGAG	hsa-miR-28-5p	MIMAT0000085
000456_hsa-miR-130b	CAGUGCAAUGAUGAAAGGGCAU	hsa-miR-130b-3p	MIMAT0000691
002271_hsa-miR-185	UGGAGAGAAAGGCAGUUCCUGA	hsa-miR-185-5p	MIMAT0000455
000475_hsa-miR-152	UCAGUGCAUGACAGAACUUGG	hsa-miR-152-3p	MIMAT0000438
000397_hsa-miR-21	UAGCUUAUCAGACUGAUGUUGA	hsa-miR-21-5p	MIMAT0000076
000507_hsa-miR-203	GUGAAAUGUUUAGGACCACUAG	hsa-miR-203a-3p	MIMAT0000264
001090_mmu-miR-93	CAAAGUGCUGUUCGUGCAGGUAG	mmu-miR-93-5p	MIMAT0000540
000408_hsa-miR-27a	UUCACAGUGGCUAAGUUCCGC	hsa-miR-27a-3p	MIMAT0000084
001515_hsa-miR-660	UACCCAUUGCAUAUCGGAGUUG	hsa-miR-660-5p	MIMAT0003338
000442_hsa-miR-106b	UAAAGUGCUGACAGUGCAGAU	hsa-miR-106b-5p	MIMAT0000680
000403_hsa-miR-25	CAUUGCACUUGUCUCGGUCUGA	hsa-miR-25-3p	MIMAT0000081
000494_hsa-miR-195	UAGCAGCACAGAAAUAUUGGC	hsa-miR-195-5p	MIMAT0000461
002276_hsa-miR-222	AGCUACAUCUGGCUACUGGGU	hsa-miR-222-3p	MIMAT0000279
000564_hsa-miR-375	UUUGUUCGUUCGGCUCGCGUGA	hsa-miR-375-3p	MIMAT0000728
002446_hsa-miR-28-3p	CACUAGAUUGUGAGCUCCUGGA	hsa-miR-28-3p	MIMAT0004502
000545_hsa-miR-331	GCCCCUGGGCCUAUCCUAGAA	hsa-miR-331-3p	MIMAT0000760
002349_hsa-miR-574-3p	CACGCUCAUGCACACACCCACA	hsa-miR-574-3p	MIMAT0003239
000470_hsa-miR-148a	UCAGUGCACUACAGAACUUUGU	hsa-miR-148a-3p	MIMAT0000243
000528_hsa-miR-301	CAGUGCAAUAGUAUUGUCAAAGC	hsa-miR-301a-3p	MIMAT0000688
002258_hsa-miR-340	UUAUAAAGCAAUGAGACUGAUU	hsa-miR-340-5p	MIMAT0004692
000563_hsa-miR-374	UUAUAAUACAACCUGAUAAGUG	hsa-miR-374a-5p	MIMAT0000727
002324_hsa-miR-744	UGCGGGGCUAGGGCUAACAGCA	hsa-miR-744-5p	MIMAT0004945
000420_hsa-miR-30d	UGUAAACAUCCCCGACUGGAAG	hsa-miR-30d-5p	MIMAT0000245
000387_hsa-miR-10a	UACCCUGUAGAUCCGAAUUUGUG	hsa-miR-10a-5p	MIMAT0000253
001097_hsa-miR-146b	UGAGAACUGAAUUCCAUAGGCUG	hsa-miR-146b-5p	MIMAT0002809
000417_hsa-miR-30a-5p	UGUAAACAUCCUCGACUGGAAG	hsa-miR-30a-5p	MIMAT0000087
000493_hsa-miR-194	UGUAACAGCAACUCCAUGUGGA	hsa-miR-194-5p	MIMAT0000460
002187_hsa-miR-942	UCUUCUCUGUUUUGGCCAUGUG	hsa-miR-942-5p	MIMAT0004985
002161_hsa-miR-324-3p	ACUGCCCCAGGUGCUGCUGG	hsa-miR-324-3p	MIMAT0000762
002367_hsa-miR-193b	AACUGGCCCUCAAAGUCCCGCU	hsa-miR-193b-3p	MIMAT0002819
002883_hsa-miR-1274A	GUCCCUGUUCAGGCGCCA	hsa-miR-1274A	MI0006410
002893_hsa-miR-1247	ACCCGUCCCGUUCGUCCCCGGA	hsa-miR-1247-5p	MIMAT0005899
000546_hsa-miR-335	UCAAGAGCAAUAACGAAAAAUGU	hsa-miR-335-5p	MIMAT0000765
002277_hsa-miR-320	AAAAGCUGGGUUGAGAGGGCGA	hsa-miR-320a-3p	MIMAT0000510
000539_hsa-miR-324-5p	CGCAUCCCCUAGGGCAUUGGUG	hsa-miR-324-5p	MIMAT0000761
000464_hsa-miR-142-3p	UGUAGUGUUUCCUACUUUAUGGA	hsa-miR-142-3p	MIMAT0000434
001020_hsa-miR-365	UAAUGCCCCUAAAAAUCCUUAU	hsa-miR-365a-3p	MIMAT0000710

Supplementary Table 4. Details of miRNAs that were differentially expressed after SLC7A11 knockdown.

Open Array identifier	miRBase v22 sequence	miRBase v22 Identifier	miRBase Accession number
000417_hsa-miR-30a-5p	UGUAAACAUCCUCGACUGGAAG	hsa-miR-30a-5p	MIMAT0000087
002883_hsa-miR-1274A	GUCCCUGUUCAGGCGCCA	hsa-miR-1274A	MI0006410
002884_hsa-miR-1274B	UCCCUGUUCGGGCGCCA	hsa-miR-1274B	MI0006427
000545_hsa-miR-331	GCCCCUGGGCCUAUCCUAGAA	hsa-miR-331-3p	MI0000812

Supplementary Figure 2. Correlations in normalised miRNA expression between the average of each p53-parental cell line vs. the pooled average of all p53-parental cell lines in each experiment.

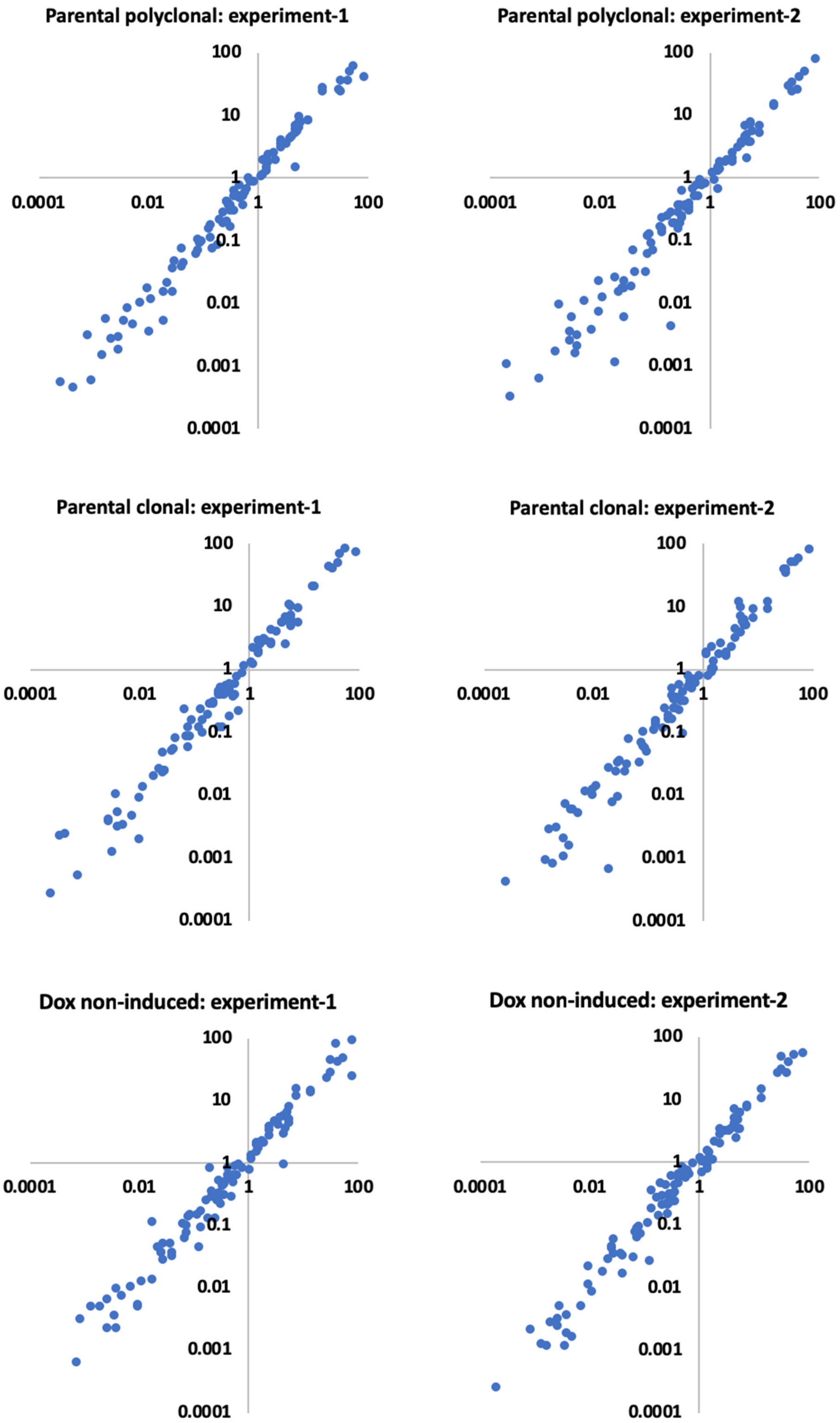

Supplementary Figure 3. Correlations in normalised miRNA expression between each p53-KO cell line and the average of all p53-KO cell lines in each experiment.

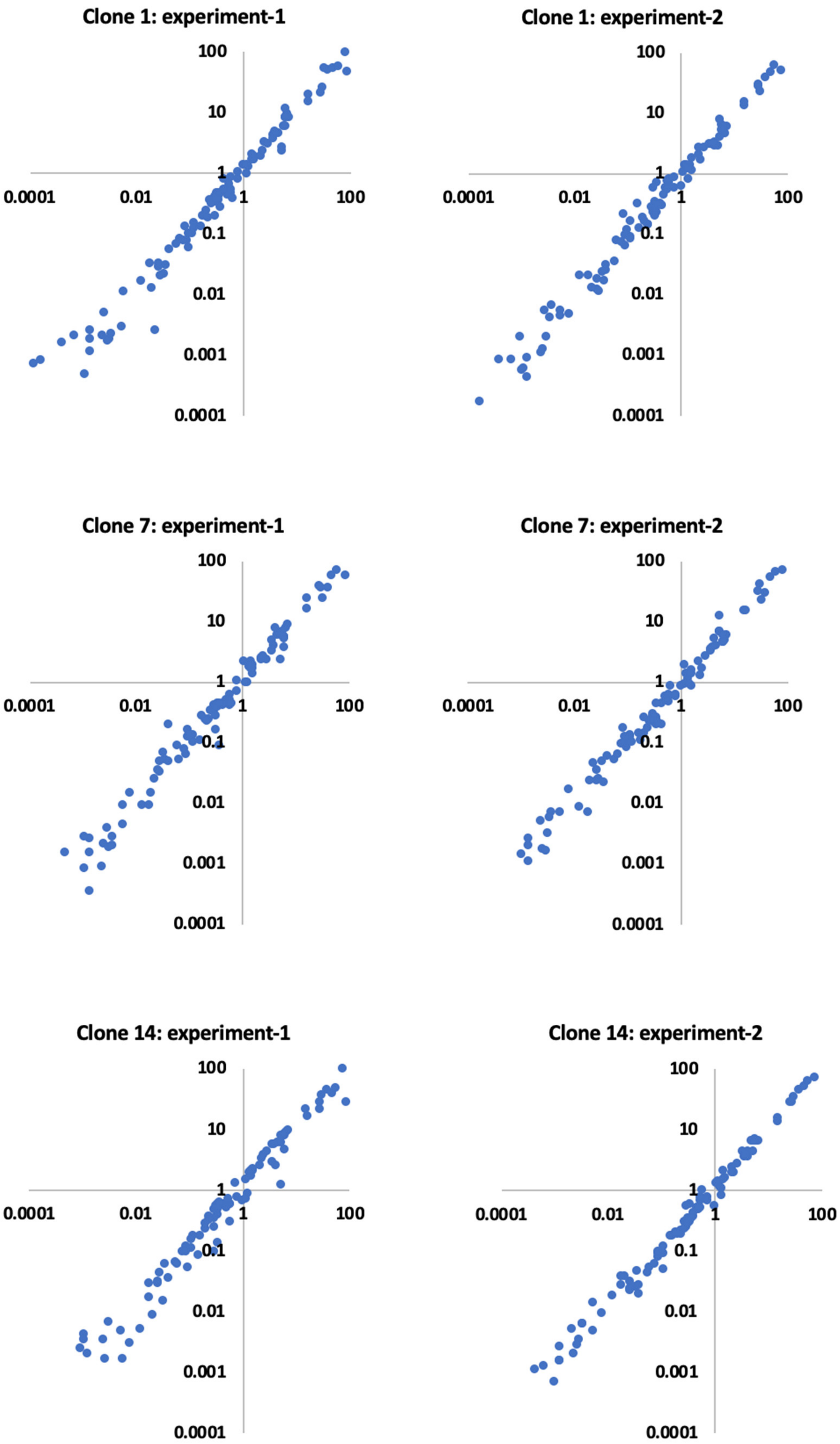

Supplementary Figure 4. Correlations in normalised miRNA expression between each p53-KO cell line experiment and the average of both experiments, for both the cells that were treated with the non-binding control and the cells treated with the siRNA that was used to knockdown SLC7A11.

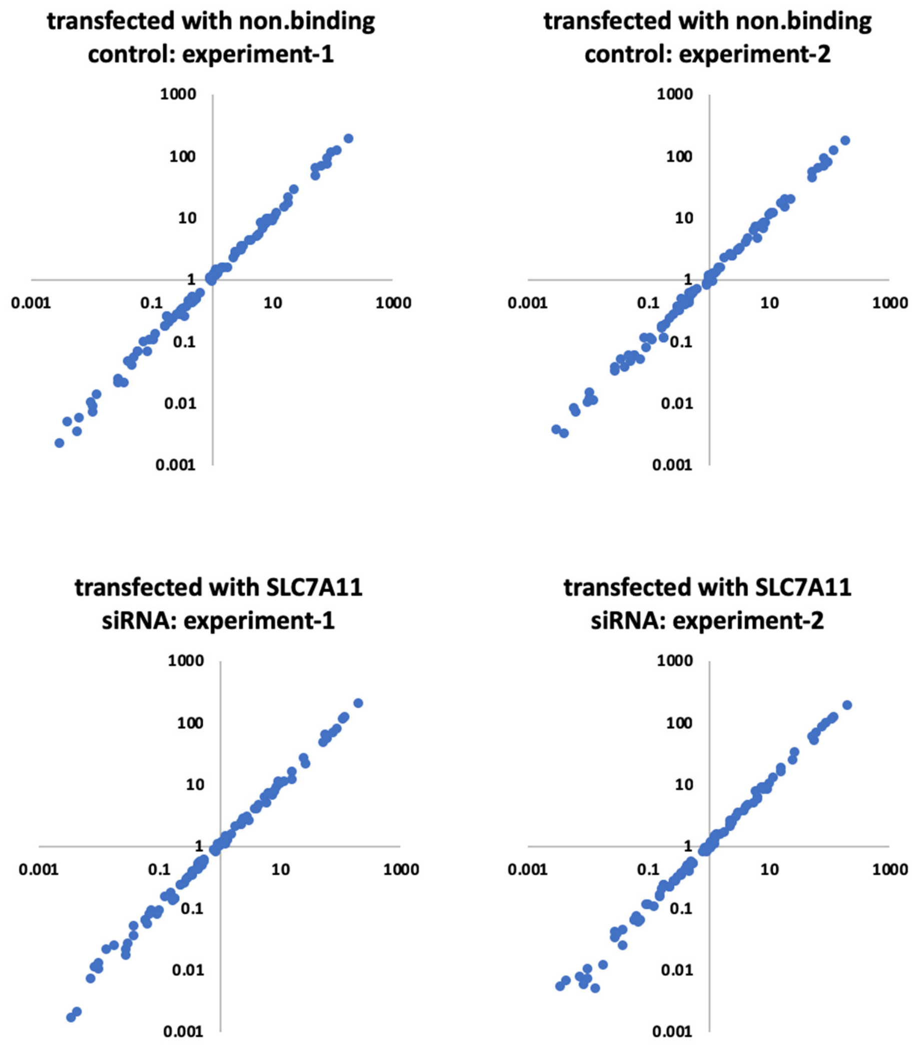

Supplement: Supplementary file 1 [file ijms-22-05547-s001.zip › ijms-1186325-supplementary/ijms-1186325 supplementary.v4/Supplementary Data revised/S3 dose response curve, details of (HKG) miRNAs, correlations in normalised miRNA expression .pdf]
