# Supplementary material for: Mutant p53 Mediates Sensitivity to Cancer Treatment Agents in Oesophageal Adenocarcinoma Associated with MicroRNA and SLC7A11 Expression
Source: Int J Mol Sci. 2021 May 24;22(11):5547. doi: 10.3390/ijms22115547 (PMC8197322; doi:10.3390/ijms22115547)
Supplement: Supplementary file 1 [file ijms-22-05547-s001.zip › ijms-1186325-supplementary/ijms-1186325 supplementary.v4/Supplementary Data revised/Western Blots revised.pdf]

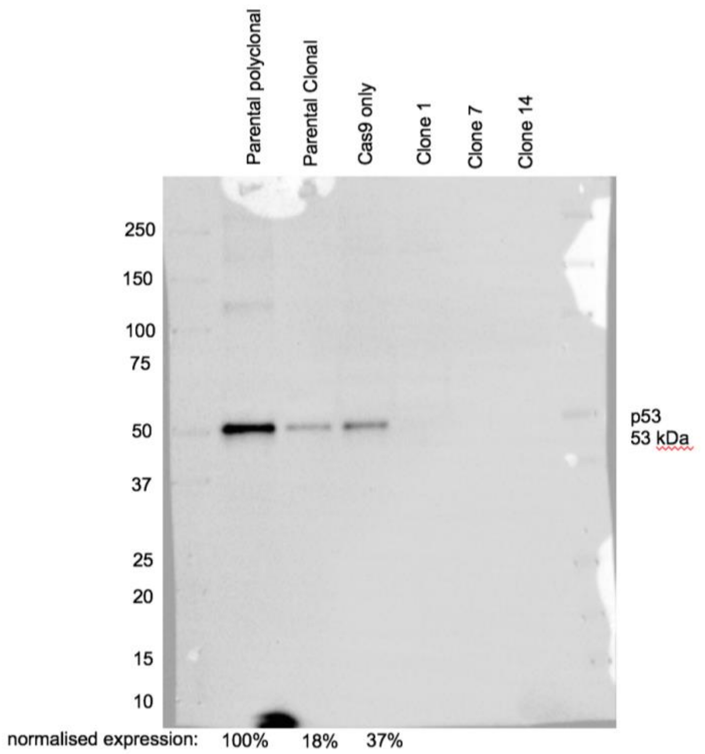

Figure 1. p53 Western Blot p53 (53 kDa) Western Blot demonstrating p53 absence in the p53-KO cells (Clone 1, Clone 7 and Clone 14). The % value represent the normalised expression (for protein quantification, protein expression was normalised to total protein load).

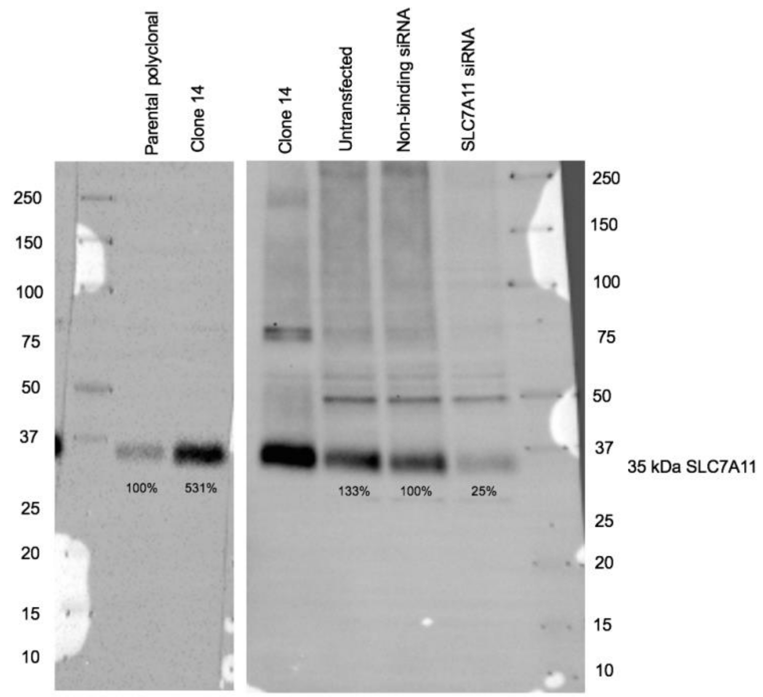

Figure 2 and Figure 3. SLC7A11 (35kDa) Western Blot

Left: SLC7A11 expression is higher in Clone 14 (p53-KO) than in Parental polyclonal (Parental).

Right: Western Blot results confirm SLC7A11 knockdown. SLC7A11 knockdown of pooled p53-KOs cells (Clone 1, 7 and 14) resulted in 75% reduction in the level of SLC7A11 expression. The % value represent the normalised expression (for protein quantification, protein expression was normalised to total protein load).

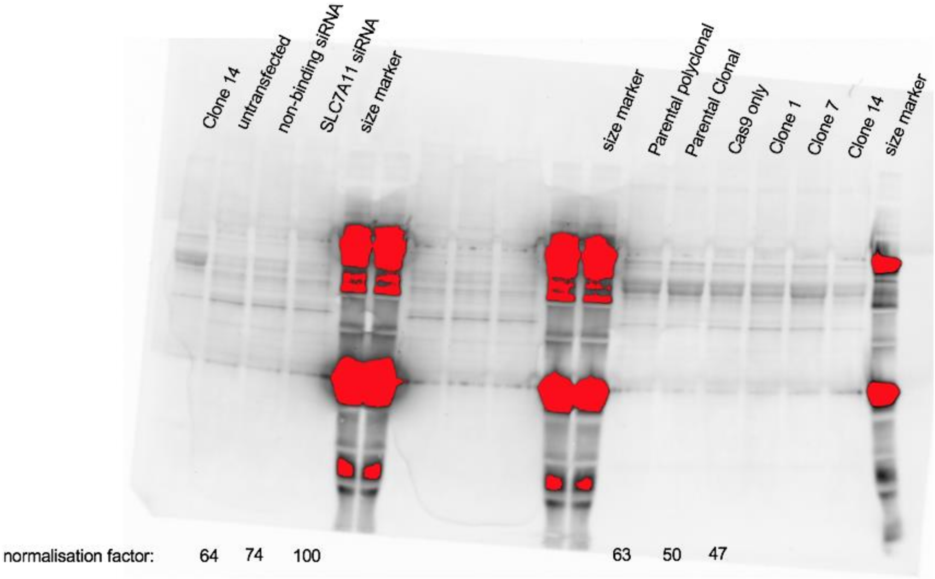

Figure 4. Protein loading control image (referring to Figure 1 and Figure 3)

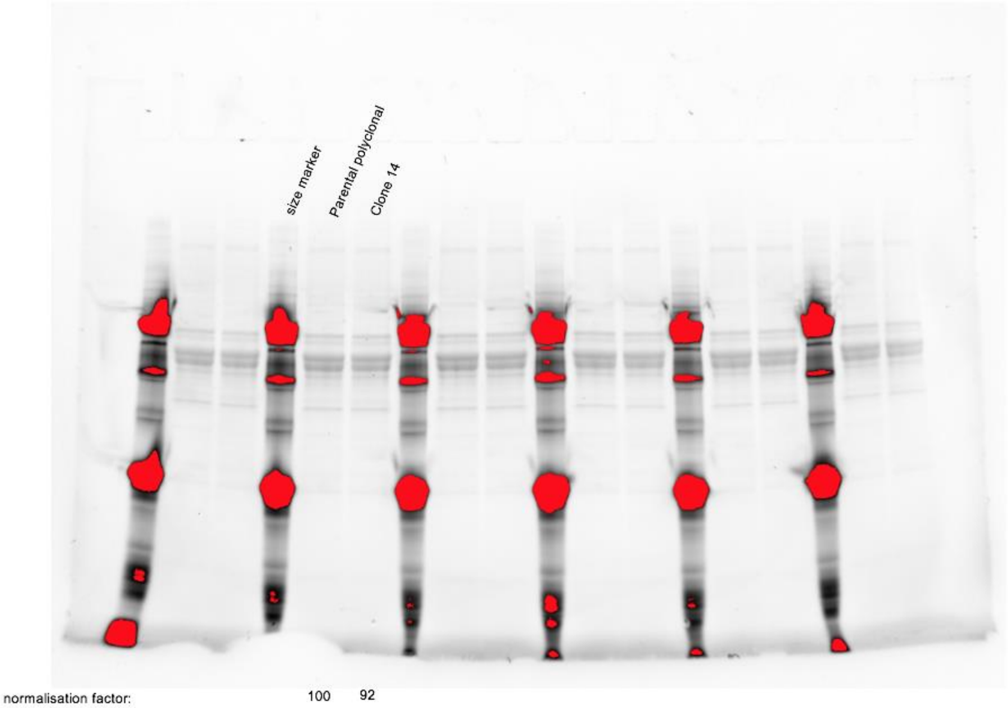

Figure 5. Protein loading control image (referring to Figure 2)
